# Reference ranges for standard-echocardiography in pugs and impact of clinical severity of Brachycephalic Obstructive Airway Syndrome (BOAS) on echocardiographic parameters

**DOI:** 10.1186/s12917-022-03348-8

**Published:** 2022-07-20

**Authors:** Pia Saskia Wiegel, Ingo Nolte, Rebekka Mach, Fritjof Freise, Jan-Peter Bach

**Affiliations:** 1 Clinic for Small Animals, University of Veterinary Medicine Hannover, Bünteweg 9, 30559 Hannover, Germany; 2 Institute for Biometry, Epidemiology and Information Processing, University of Veterinary Medicine Hannover, Bünteweg 2, 30559 Hannover, Germany

**Keywords:** Brachycephaly, Canine, Cardiac ultrasound, Cardiovascular, Reference values

## Abstract

**Background:**

Echocardiographic measurements may be influenced by breed-specific characteristics. Therefore, this study aims to establish reference values for standard echocardiographic measurements in pugs by investigating the influence of age, sex, heart rate, body weight and Brachycephalic Obstructive Airway Syndrome (BOAS). Sixty-two privately owned pugs underwent physical examination, blood sample collection, non-invasive blood pressure measurements and echocardiography. Influences of independent variables on echocardiographic measurements were examined using a multiple linear regression analysis model. For the entire study population, 95% prediction intervals were generated. Further, reference ranges for subcategories of clinical severities of BOAS were provided. Selected echocardiographic measurements of pugs were compared to reference values of previous studies generated from various breeds.

**Results:**

In the study, a total of fifty-one privately owned pugs aged between two and 10 years were included for establishing reference ranges. Mainly body weight, but also age, sex and heart rate had influence on several echocardiographic parameters. The clinical grading of BOAS was conducted in 42 pugs. Except for pulmonic peak velocity (Pvel), which declined with increasing severity of BOAS, clinical symptoms of upper airway disease did not have significant impact on echocardiographic measurement results. Significant deviations, however, of left ventricular (LV) internal dimension (LVID), interventricular septum (IVS), LV posterior wall (LVPW), and tricuspid annular plane systolic motion excursion (TAPSE) compared to interbreed reference values were observed.

**Conclusions:**

Breed-specific reference ranges for echocardiographic values with special regard to BOAS are provided to enable a more accurate assessment of cardiac health in pugs.

**Supplementary Information:**

The online version contains supplementary material available at 10.1186/s12917-022-03348-8.

## Background

Echocardiography is indispensable for diagnostic investigation and monitoring of cardiovascular diseases in dogs. For differentiation between structurally normal and abnormal cardiac anatomy, echocardiographic reference values are necessary [1, 2].

Due to breed-specific and individual differences in dog size and anatomy, absolute reference values are often not applicable. More diagnostically conclusive values can be obtained by normalising measurements to body weight (BW) or body surface area (BS) [3, 4]. Normalisation can also be achieved by forming ratios with other cardiac structures, such as the aorta [5–7]. In most cases, reference values are initially determined, based on measurements acquired in a population of different breeds [4, 8–10].

While using normalised reference values in diagnostic imaging of the heart is an important step towards objective assessment of cardiac health, different radiographic and ultrasonographic studies have shown that there can be significant differences between breeds [11, 12]. Due to this, investigation of breed-specific values for cardiac imaging in dogs and comparison to commonly used interbreed reference ranges are needed. Thus, specific reference ranges for echocardiography have already been established for various breeds. For example, studies exist for the Border Collie, the Cavalier King Charles Spaniel, the English Springer Spaniel, the Leonberger, the Saluki, the Whippet and many more [13–19]. The pug currently lacks specific references for echocardiography, even though its distinctive body traits such as the compact body conformation with a barrel chest and the most striking characteristic, that of brachycephaly, might make establishing breed-specific reference values beneficial.

Brachycephaly often leads to the well-known respiratory disease of the upper airway tract, the Brachycephalic Obstructive Airway Syndrome (BOAS) [20–24]. Affected dogs may present a variety of clinical signs, from mild respiratory symptoms such as noisy breathing up to dyspnea and severe impairment of physical fitness and heat intolerance [25, 26]. Pugs show a high prevalence for clinical impairment by BOAS [27]. Chronic, obstructive respiratory diseases, such as BOAS can influence the cardiovascular system and may result in secondary changes, for example pulmonary hypertension (PH) [28–30]. Therefore, the influence of the severity of BOAS and its impact on echocardiographic parameters is of a special interest. Only few studies on echocardiography in brachycephalic dogs exist [31, 32] and in these, the pug is underrepresented. Even though comparisons to mesocephalic control dogs have been conducted in these studies, there are currently no studies investigating echocardiographic differences with a focus on varying clinical impairment of BOAS.

Therefore, the present study aims to establish reference values for standard echocardiographic measurements for pugs and to identify influences of heart rate (HR), BW, sex, age and presence of various forms of BOAS on echocardiographic variables. Additionally, a selection of echocardiographic values of pugs were compared to interbreed reference values.

## Methods

### Animals and examinations

Sixty-two privately-owned pugs with an age of at least 24 months were prospectively examined at the Clinic for Small Animals of the University of Veterinary Medicine Hannover, Hannover, Germany. Owners signed an informed consent agreeing to all examinations. The Lower Saxony State Office for Consumer Protection and Food Safety (LAVES) approved the study (file no. 33.19-42502-05-19A424).

Each dog underwent recording of case history, a physical examination, blood sample collection (haematology and biochemistry), non-invasive blood pressure measurements and echocardiographic examination (details below). Non-invasive blood pressure measurements were performed at the end of the study day in a quiet environment employing a high-definition-oscillometry device (HDO Pro, S+B MedVet GmbH, Babenhausen, Germany) in accordance with the manufacturer and using the same protocol for each patient. Echocardiographic reference ranges were determined using all patients that did not show any signs of cardiac, vascular or any other significant systemic disease (except for BOAS) in either of the abovementioned examinations, and these dogs were used for comparison to interbreed studies. In detail, dogs were excluded from consideration if they showed one or more of the following criteria: a) pathological heart murmur, gallop sound or nonsinus-arrhythmia; b) current or recent evidence of a severe systemic illness or medications affecting the cardiovascular system based on history or physical examination (except for BOAS); c) abnormal, non-invasive blood pressure (systolic arterial pressure (SAP) > 160 mmHg) d), distinct lack of cooperation during echocardiographic examination; or e) significant cardiac abnormalities identified on two-dimensional (2-D), M-Mode, and/or Doppler mode (e. g. congenital structural defects). Minor valve regurgitations not audible via auscultation, without any structural valve abnormalities and jet area to left atrium-percentage of less than 15% assessed via colour Doppler were considered clinically non-significant and these dogs were not excluded from the study [18, 33].

Additionally, a clinical assessment of BOAS was performed during physical examination and in an advanced step of the study, using an established functional grading-system utilising an exercise test (ET) [26, 27], slightly modified to match the present study design. Each subject was introduced to the ET, which was performed on a treadmill at an individual trotting pace at a later point in time on the study day, at least one hour after the echocardiographic examination. According to the results of this functional grading system, pugs that completed the ET could be graded into non-affected (Grade 0), mildly affected (Grade 1), moderately affected (Grade 2) and severely affected (Grade 3) by BOAS. Furthermore, Grades 0 and 1 were summarised as a clinically non-affected (BOAS-) and Grades 2 and 3 as clinically affected (BOAS+) group [26]. A more detailed description of the functional grading system can be found in supplementary materials (Additional File 1). Dogs that were unable to complete the ET due to a lack of cooperation on the treadmill could not be evaluated completely regarding the above-mentioned functional grading system. They were therefore excluded from the comparison of echocardiographic measurements in relation to BOAS.

### Echocardiography

Echocardiographic examination was performed by one experienced examiner (JPB) using a GE Vivid 9 or 7 (GE Healthcare GmbH, Solingen, Germany). Simultaneous lead II Electrocardiogram was recorded. If any abnormalities were detected in continuous ECG during echocardiographic examination, dogs underwent an additional 3 lead ECG. Dogs were examined in right and left lateral recumbency in a quiet, dark room without sedation. The same standardised imaging protocol was used for each examination. Images were acquired in accordance with published guidelines [34]. Variables obtained in standard 2-D and M-Mode were measured following the recommendations set by the American Society of Echocardiography and published methodology in the veterinary literature [6, 35–40]. An average HR was generated from 3 to 5 R-R intervals in right recumbency mid-examination. Detailed description of echocardiographic measurements can be found in supplementary material (Additional File 1).

### Comparison to interbreed studies

Comparison of selected echocardiographic measurements in pugs to values of a selection of existing studies deriving from multiple breeds was conducted (Table 1). The following factors were considered for selection: a proper variety of breeds (> 10 breeds, if possible; mixed breeds or unknown breeds were included), a large amount of subjects (> 120 subjects of various breeds per study), year of publication not older than 20 years, development of an allometric scaling for standard LV M-Mode measurements and comparable execution of measurements of echocardiographic parameters.

**Table 1:** Tabular summary of the selection for comparison of interbreed studies to values of pugs

**Author and year of publication**	**comparison parameters**
Cornell et al. 2004 [4]	LVIDd/s, IVSd/s, LVPWd/s
Visser et al. 2015 [41]	TAPSE
Caivano et al. 2018 [42]	TAPSE:Ao
Rishniw et al. 2019 [8]	LA:Ao
Visser et al. 2019 [9]	LVIDd/s, LA:Ao
Esser et al. 2020 [10]	LVIDd/s, IVSd/s, LVPWd/s

*IVSd/s* Interventricular septum diastole/systole, *LA:Ao* Left-atrial-to-aortic-root diameter, *LVIDd/s* Left ventricular internal diameter diastole/systole, *LVPWd/s* Left ventricular posterior wall diastole/systole, *TAPSE* Tricuspid annular plane systolic motion excursion

For comparison of standard M-Mode measurements, the developed allometric scaling exponents of each interbreed study were applied on measurements of this study population, thus normalising M-Mode measurements of pugs with the equivalent exponents. These normalised measurements of pugs were then compared to the equivalent interbreed reference values of each publication.

### Statistical analyses

Data were statistically analysed using SAS-Software (Version 9.4, SAS Institute Inc., Cary, NC, USA). Values of *p* < 0.05 were considered statistically significant. Outlier analysis was performed by visual analysis and as described in the Guidelines for the Determination of Reference Intervals in Veterinary Species [43]. All variables were tested for normal distribution using the Shapiro-Wilk test, and visual examination using histograms and Q-Q-plots. Pearson or Spearman correlation coefficients between independent variables (BW, age, HR) and echocardiographic values were calculated. Comparison of independent variables between males and females was performed using the t-test.

#### Establishment of reference values and comparison of BOAS subgroups/Grades

Multiple linear regression analyses were performed to examine linear relationship between echocardiographic measurements and four independent variables (BW, HR, age, sex). For this purpose, a backward selection for the multiple regression model was run for each echocardiographic parameter. The selection process stopped and was accepted at the resulting model when all variables in it were statistically significant. Multiple linear regression analysis equations were created for each echocardiographic parameter where at least one independent variable was statistically significant. Adjusted r-square (R^2^) is given for assessing the adequacy of fit of the model. It states the percentage of variation that can be explained by the independent variables that affect the dependent parameter [44]. A maximum R^2^ of 1 would mean that 100% of the observed variation can be explained by the model’s input. If R^2^ increases with additional input variables, these variables add more value to the model. For the entire study population included in the analysis, 95% prediction intervals using the samples quantiles (2.5% and 97.5% limits) were generated. For each lower and upper limit of prediction interval, 90% confidence intervals (CI) were computed using the bootstrap method. Linear regression analyses were also used to create weight-based prediction values and 95% prediction intervals for selected variables, where only body weight was statistically significant.

Overall comparison of demographic and echocardiographic measurements of different BOAS Grades (Grades 0 to 3) was performed with the Kruskal-Wallis test. Additionally, comparison of measurements between BOAS- and BOAS+ group was conducted using the Wilcoxon rank-sum test.

#### Comparison to reference ranges of pugs to those of other studies

Reference values of a selection of interbreed studies were compared to measurements for pugs and, if applicable, the given allometric equations corresponding to each publication were used. For comparison of reference values of pugs with those of other interbreed studies, either a sign test (if median was specified) or a t-test (if mean was specified) was used.

## Results

### Establishment of references ranges and comparison of BOAS subgroups and Grades

**Fig. 1:**
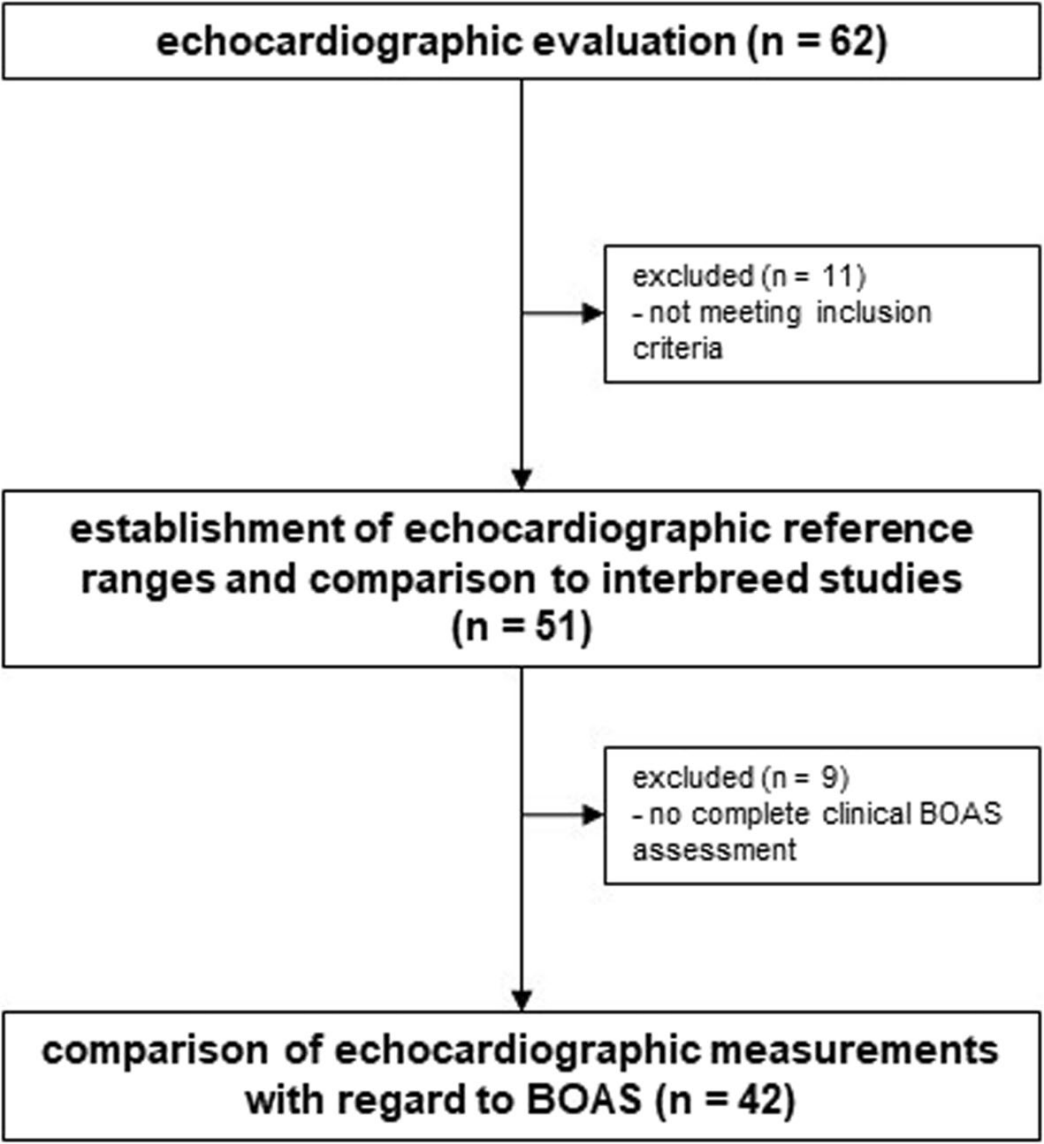
Assessment for eligibility

A total of 62 pugs were eligible for inclusion in the present study and received echocardiographic evaluation and were introduced to the ET. Five pugs were excluded from the study due to non-sinus arrhythmia (*n* = 4 second degree atrioventricular block, *n* = 1 right bundle branch block), three showed valve regurgitations that exceeded predetermined criteria (*n* = 1 mitral regurgitation, *n* = 1 tricuspid regurgitation and *n* = 1 pulmonic regurgitation, all moderate). Another three animals could not be examined due to a lack of cooperation during echocardiography. Thus, a total of 51 pugs met the defined criteria for establishing reference ranges and were included in further investigations. Out of these 51 pugs, 42 were included in comparison of echocardiographic parameters with regard to BOAS (Fig. 1). Nine pugs showed a distinct lack of cooperation on the treadmill or during auscultation throughout the ET and thus could not be evaluated completely regarding the functional grading system (lack of post-exercise evaluation). Out of these nine subjects, three dogs showed no respiratory symptoms and six at least mild respiratory symptoms irrespective of the ET (e.g. varying respiratory noise, increased inspiratory effort during physical examination).

Age, BW, BS, HR, sex, SAP and classification of BOAS of the study population for establishing the reference ranges are presented in Table 2. Regarding correlation between independent variables, significant association was found for sex, which had a significant effect on BW and BS (both *p* < 0.0001). Except for age, all independent variables were normally distributed.

**Table 2:** Demographic data of 51 pugs meeting criteria for establishing reference ranges

***N*** **= 51**	**mean ± SD**	**median**	**range** (min - max)
**age [years]**	4.5 ± 2.2	4.3	2 - 10.5
**BW [kg]**	8.9 ± 1.3	8.8	6 - 12.4
**BS [m²]**	0.43 ± 0.04	0.43	0.33 - 0.54
**HR [bpm]**	110 ± 23	105	70 - 160
**SAP [mmHg] **(*N* = 50)	119.6 ± 11.8	117.5	100 - 146
**sex**			
**male (n/i)**	24 (4/20)		
**female (n/i)**	27 (10/17)		
**BOAS** (*N* = 42)			
**Grade**	Grade 0: 31% (*N* = 13)	} BOAS- (*N* = 21)	
	Grade 1: 19% (*N* = 8)		
	Grade 2: 35,7% (*N* = 15)	} BOAS+ (*N* = 21)	
	Grade 3: 14,3% (*N* = 6)		

*bpm* beats per minute, *BOAS* Brachycephalic Obstructive Airway Syndrome, *BS* body surface area, *BW* body weight, *HR* heart rate, *i* intact, *max* maximum, *min* minimum, *N* number of subjects, *n* neutered, *SAP* systolic arterial blood pressure, *SD* standard deviation

Note: BOAS functional grading system according to Liu et al. 2015, modified to present study design

Out of the 51 pugs included in the study for reference ranges, demographic data were further allocated to each BOAS Grade 0 to 3 and BOAS-/+ for comparison of subgroups in all pugs that completed the clinical assessment (*n* = 42) (Table 3). When comparing demographic variables (age, BW, BS, HR, SAP and sex) of BOAS- and BOAS+ groups and BOAS Grades 0 to 3, no significant differences could be detected between the groups and Grades. Demographic data for pugs divided into BOAS Grades can be found in supplementary material (Additional File 2).

**Table 3:** Mean ± SD of demographic data for 42 pugs allocated to BOAS groups

	**BOAS-**	**BOAS+**	***p*** ** value**
*N* = 42	21	21	
**age [years]**	4.1 ± 2.0	4.6 ± 2.2	0.5164
**BW [kg]**	9.0 ± 1.2	8.7 ± 1.4	0.5489
**BS [m**^**2**^**]**	0.44 ± 0.04	0.43 ± 0.05	0.5311
**HR [bpm]**	107 ± 22	113 ± 21	0.5305
**SAP [mmHg]**	118.2 ± 11.9	121.1 ± 12.1	0.3884
**sex**			
** male (** ***n*** **/i)**	10 (1/9)	10 (2/8)	1.0000
** female (** ***n*** **/i)**	11 (2/9)	11 (5/6)	

*BOAS* Brachycephalic Obstructive Airway Syndrome, *bpm* Beats per minute, *BS* Body surface area, *BW* Body weight, *HR* Heart rate, *i* Intact, *N* Number of subjects, *n* Neutered, *SAP* Systolic arterial pressure

BOAS functional grading system according to Liu et al. 2015, modified to present study design. P values from comparsion of BOAS- to BOAS+ group by using Wilcoxon rank-sum test. Significant p values are highlighted with an asterisk (*).

Regarding echocardiographic measurements, the following parameters did not show normal distribution: left ventricular (LV) width in systole (LVWs), aortic root diameter (Ao), aortic peak velocity (Avel), mitral E to A wave (MV E:A), tricuspid E-wave and A-wave (TV E, TV A). Multiple linear regression equations were computed and can be found in the supplementary material (Additional File 2). All echocardiographic parameters showing significant influence in the multiple linear regression model, except for ejection fraction (EF), showed significant correlations to the independent variables, as well (Pearson or Spearman correlation coefficients).

Mean, standard deviation (SD), median, range, 95% prediction intervals and 90% CI’s for lower and upper prediction limits for all echocardiographic parameters for 51 pugs included to establish reference ranges are presented in Table 4.

**Table 4:** *2-D, M-Mode and Doppler measurements for 51 pug*s *meeting criteria for establishing reference ranges*

	**mean ± SD**	**median**	**range** (min - max)	**95% prediction interval** (90% CI’s for limits)	
**2-D measurements and SMOD-derived LV volumes**					
LA [cm]	2.01 ± 0.37	1.96	1.3 - 2.69	1.32 - 2.56	(1.3 - 1.51) - (2.52 - 2.69)
Ao [cm]	1.51 ± 0.16	1.51	1.1 - 1.95	1.1 - 1.92	(1.1 - 1.31) - (1.68 - 1.95)
LA:Ao	1.32 ± 0.17	1.3	0.95 - 1.65	0.97 - 1.63	(0.95 - 1.13) - (1.56 - 1.65)
LVLd [cm]	4.27 ± 0.44	4.2	3.49 - 5.25	3.56 - 5.22	(3.49 - 3.72) - (4.85 - 5.26)
LVWd [cm]	2.61 ± 0.27	2.62	1.95 - 3.28	2.14 - 3.03	(1.95 - 2.27) - (2.93 - 3.28)
SI	1.64 ± 0.17	1.62	1.3 - 2.17	1.33 - 1.93	(1.3 - 1.45) - (1.85 - 2.17)
LVEDV [mL]	18.76 ± 2.33	18.77	9.5 - 27.25	11.63 - 26.58	(9.5 - 13.8) - (24.02 - 27.25)
LVESV [mL]	7.35 ± 2.33	7.21	3.06 - 12.54	3.41 - 11.99	(3.06 - 4.74) - (10.60 - 12.54)
EDVI [mL/m^2^]	43.07 ± 7.99	43.27	26.61 - 57.71	27.57 - 57.69	(26.61 - 32.9) - (52.46 - 57.71)
ESVI [mL/m^2^]	16.98 ± 5.23	16.7	7.09 - 29.1	8.61 - 28.46	(7.09 - 10.97) - (23.96 - 29.1)
EDV:BW [mL/kg]	2.1 ± 0.39	2.09	1.29 - 2.88	1.32 – 2.79	(1.29 - 1.64) - (2.74 - 2.88)
ESV:BW [mL/kg]	0.83 ± 0.27	0.81	0.33 - 1.5	0.43 – 1.43	(0.33 - 0.52) - (1.19 - 1,5)
EF [%]	60.67 ± 9.16	60.46	35.9 - 82.66	45.39 - 75.28	(35.9 - 50.24) - (74.05 - 82.66)
**M-mode measurements**					
*LV measurements*					
LVIDd [cm]	2.65 ± 0.3	2.66	1.94 - 3.71	2.11 - 3.21	(1.94 - 2.28) - (2.95 - 3.71)
LVIDs [cm]	1.86 ± 0.27	1.91	1.02 - 2.43	1.46 - 2.34	(1.02 - 1.5) - (2.17 - 2.43)
IVSd [cm]	0.79 ± 0.15	0.74	0.52 - 1.11	0.55 - 1.09	(0.52 - 0.62) - (0.99 - 1.11)
IVSs [cm]	1.05 ± 0.09	1.04	0.82 - 1.48	0.82 - 1.39	(0.82 - 0.87) - (1.22 - 1.48)
LVPWd [cm]	0.8 ± 0.09	0.79	0.64 - 1.02	0.66 - 0.99	(0.64 - 0.69) - (0.92 - 1.02)
LVPWs [cm]	1.11 ± 0.14	1.1	0.86 - 1.46	0.87 - 1.41	(0.86 - 0.97) - (1.28 - 1.46)
FS [%]	29.31 ± 6.27	29.5	17.43 - 44.86	18.71 - 43.79	(17.43 - 22.82) - (37.04 - 44.86)
EPSS [mm]	2.89 ± 6.27	2.75	0.7 - 6.1	0.9 - 5.9	(0.7 - 1.5) - (4.5 - 6.1)
*RV measurements (N = 40)*					
TAPSE [mm]	9.32 ± 1.75	9.2	5.5 - 13.4	6 - 12.75	(5.5 - 7.6) - (11.5 - 13.4)
TAPSE:Ao	0.62 ± 0.11	0.61	0.34 - 0.81	0.38 - 0.81	(0.34 - 0.49) - (0.77 - 0.81)
**Doppler measurements**					
Avel [m/s]	1.39 ± 0.24	1.39	0.92 - 1.84	0.94 - 1.75	(0.92-1.05) - (1.65 - 1.84)
Pvel [m/s]	0.81 ± 0.16	0.79	0.51 - 1.25	0.54 - 1.17	(0.51 - 0.6) - (1.02 - 1.25)
MV E [m/s]	0.8 ± 0.13	0.82	0.43 - 1.13	0.62 - 1.08	(0.43 - 0.65) - (0.95 - 1.13)
MV A [m/s]	0.6 ± 0.09	0.59	0.39 - 0.82	0.42 - 0.77	(0.39 - 0.48) - (0.7 - 0.82)
MV E:A	1.36 ± 0.29	1.35	0.56 - 2.17	0.61 - 1.07	(0.56 - 1.07) - (1.71 - 2.17)
TV E [m/s]	0.69 ± 0.13	0.71	0.43 - 1.14	0.46 - 0.96	(0.43 - 0.49) - (0.8 - 1.14)
TV A [m/s]	0.5 ± 0.11	0.48	0.31 - 0.75	0.32 - 0.74	(0.31 - 0.4) - (0.67 - 0.75)
TV E:A	1.42 ± 0.34	1.49	0.68 - 2.38	0.79 - 2.09	(0.68 - 0.93) - (1.71 - 2.38)

*2-D* Two-dimensional, *Ao* Aortic root diameter, *Avel* Aortic peak velocity, *CI* Confidence interval, *EF* Ejection fraction, *EDV:BW* Indexed left ventricular end-diastolic volume to body weight, *EDVI* Indexed left ventricular end-diastolic volume to body surface area, *ESV:BW* Indexed left ventricular end-systolic volume to body weight, *ESVI* Indexed left ventricular end-systolic volume to body surface area, *EPSS* E-Point to septum separation, *FS* Fractional shortening, *IVSd/s* Interventricular septum diastole/systole, *LA* Left atrial diameter, *LV* Left ventricle, *LVEDV* Left ventricular end-diastolic volume, *LVESV* Left ventricular end-systolic volume, *LVIDd/s* Left ventricular internal diameter diastole/systole, *LVLd* Left ventricular length diastole, *LVPWd/s* Left ventricular posterior wall diastole/systole,* LVWd* Left ventricular width diastole, *MV A* Mitral A-wave, *MV E* Mitral E-wave, *Pvel* Pulmonic peak velocity, *RV* Right ventricle, *SI* Sphericity index, *SD* Standard deviation, *SMOD* Simpson’s modified method of discs, *TAPSE* Tricuspid annular plane systolic motion excursion, *TV A* Tricuspid A-wave, *TV E* tricuspid E-wave

Echocardiographic measurements of the study population were allocated to each BOAS Grade 0 to 3 and BOAS-/+ for comparison of subgroups in all pugs that completed clinical assessment (*n* = 42). Mean, SD, median and range of BOAS+/- groups (Table 5) were stated. Complete data for measurements of each BOAS Grade can be found in supplementary material (Additional File 2).

**Table 5:** Echocardiographic measurements of 42 pugs subdivided into BOAS- and BOAS+ group

	**BOAS-**			**BOAS+ **		
N = 42	21			21		
	**mean ± SD **	**median**	**range** ** (min - max) **	**mean ± SD **	**median **	**range** ** (min - max)**
**2-D measurements and SMOD-derived LV volumes**						
LA [cm]	2.07 ± 0.37	1.99	1.33 - 2.69	1.97 ± 0.35	1.95	1.42 - 2.56
Ao [cm]	1.52 ± 0.13	1.51	1.22 - 1.7	1.53 ± 0.18	1.5	1.25 - 1.95
LA:Ao	1.36 ± 0.18	1.37	0.95 - 1.63	1.29 ± 0.17	1.24	0.97 - 1.61
LVLd [cm]	4.28 ± 0.4	4.27	3.61 - 5.26	4.23 ± 0.52	4.18	3.49 - 5.22
LVWd [cm]	2.64 ± 0.26	2.7	2.14 - 2.97	2.58 ± 0.3	2.62	1.95 - 3.28
SI	1.64 ± 0.17	1.61	1.3 - 1.93	1.66 ± 0.2	1.64	1.33 - 2.17
LVEDV [mL]	19.15 ± 4.32	19.81	11.63 - 26.33	18.03 ± 3.99	18.41	9.5 - 27.25
LVESV [mL]	7.41 ± 2.39	6.75	3.7 - 11.69	7.21 ± 1.87	7.21	3.41 - 11.99
EDVI [mL/m²]	43.93 ± 9.75	44.3	26.61 - 57.71	42.05 ± 6.9	41.94	27.57 - 52.46
ESVI [mL/m²]	17.17 ± 6.15	14.8	8.73 - 29.1	16.97 ± 4.12	16.76	7.09 - 25.7
EDV:BW [mL/kg]	2.14 ± 0.5	2.18	1.29 - 2.88	2.07 ± 0.34	2.05	1.51 - 2.63
ESV:BW [mL/kg]	0.84 ± 0.32	0.71	0.43 - 1.5	0.84 ± 0.22	0.82	0.33 - 1.31
EF [%]	61.03 ± 10.26	61.32	35.9 - 74.51	59.48 ± 7.76	58.53	49.81 - 82.66
**M-mode measurements**						
*LV measurements*						
LVIDd [cm]	2.67 ± 0.22	2.71	2.11 - 3.1	2.56 ± 0.27	2.59	1.94 - 3.19
LVIDs [cm]	1.84 ± 0.29	1.84	1.02 - 2.24	1.84 ± 0.26	1.82	1.46 - 2.43
IVSd [cm]	0.78 ± 0.13	0.8	0.57 - 1.09	0.82 ± 0.2	0.82	0.55 - 1.28
IVSs [cm]	1.03 ± 0.12	1.02	0.82 - 1.25	1.07 ± 0.19	1.08	0.82 - 1.48
LVPWd [cm]	0.8 ± 0.10	0.8	0.66 - 1.02	0.8 ± 0.08	0.82	0.66 - 0.94
LVPWs [cm]	1.13 ± 0.13	1.11	0.89 - 1.41	1.04 ± 0.18	1.05	0.44 - 1.34
FS [%]	30.26 ± 6.7	30.69	17.43 - 41.22	28.06 ± 6.32	26.32	19.09 - 44.86
EPSS [mm]	3.14 ± 1.97	2.6	1.3 - 10.1	3 ± 1.36	3	0.7 - 6.1
*RV measurements *	*(N = 17)*			*(N = 16)*		
TAPSE [mm]	9.55 ± 1.31	9.7	7.1 - 12.1	9.88 ± 1.96	9.2	6.8 - 13.4
TAPSE:Ao	0.62 ± 0.08	0.62	0.47 - 0.75	0.65 ± 0.12	0.62	0.48 - 0.81
**Doppler measurements**						
Avel [m/s]	1.4 ± 0.26	1.31	0.92 - 1.84	1.39 ± 0.24	42	0.97 - 1.75
Pvel [m/s]	0.86 ± 0.17	0.85	0.51 - 1.17	0.76* ± 0.11	0.78	0.54 - 0.92
MV E [m/s]	0.82 ± 0.12	0.82	0.62 - 1.08	0.82 ± 0.14	0.78	0.64 - 1.13
MV A [m/s]	0.63 ± 0.14	0.61	0.48 - 1.15	0.59 ± 0.08	0.58	0.43 - 0.73
MV E:A	1.33 ± 0.24	1.36	0.61 - 1.71	1.4 ± 0.32	1.29	1.03 - 2.17
TV E [m/s]	0.71 ± 0.12	0.72	0.43 - 0.9	0.7 ± 0.15	0.69	0.48 - 1.14
TV A [m/s]	0.51 ± 0.14	0.49	0.18 - 0.75	0.49 ± 0.1	0.48	0.35 - 0.71
MV E:A	1.53 ± 0.68	1.42	0.84 - 3.94	1.47 ± 0.36	1.49	0.79 - 2.38

*significantly lower than BOAS- (*p* = 0.0401)

*2-D* two-dimensional, *Ao* aortic root diameter *Avel* aortic peak velocity, *BOAS* Brachycephalic Obstructive Airway Syndrome *EF* ejection fraction, *EDV:BW* left ventricular end-diastolic volume indexed to body weight, *EDVI* indexed left ventricular end-diastolic volume to body surface area, *ESV:BW* left ventricular end-systolic volume indexed to body weight, *ESVI* indexed left ventricular end-systolic volume to body surface area, *EPSS* E-Point to septum separation, *FS * fractional shortening, *IVSd/s* interventricular septum diastole/systole, *LA * left atrial diameter, *LV* left ventricle, *LVEDV* left ventricular end-diastolic volume, *LVESV* left ventricular end-systolic volume, *LVIDd/s* left ventricular internal diameter diastole/systole, *LVLd* left ventricular length diastole, *LVPWd/s* left ventricular posterior wall diastole/systole, *LVWd* left ventricular width diastole, *MV A* mitral A-wave, *MV E* mitral E-wave, *N * number of subjects, *Pvel* pulmonic peak velocity, *RV* right ventricle *SI * sphericity index, *SD* standard deviation, *SMOD* Simpson’s modified method of discs, *TAPSE* tricuspid annular plane systolic motion excursion (*n* = 33), *TV A* tricuspid A-wave, *TV E* tricuspid E-wave

No significant differences between echocardiographic measurements of BOAS+ and BOAS- groups could be detected, except for pulmonic peak velocity (Pvel), which was found to be significantly lower in BOAS+ than BOAS- (*p* = 0.0401).

When examining the BOAS Grades, no significant differences of echocardiographic measurements between Grades could be detected.

### Comparison to a selection of published interbreed studies

After normalisation of M-Mode measurements of pugs from the present study with the corresponding allometric exponents of each interbreed study, significant differences between medians could be observed for various parameters (Table 6). An overview of the percentage of pugs deviating from the reference intervals (RI) of the compared interbreed studies can be found in supplementary material (Additional File 2).

**Table 6:** M-Mode values of three interbreed publications compared to normalised values from the present study

		**median and RI of interbreed study**		**exponent**	scaled median and RI of pugs (*N* = 51)		*p* value
Cornell et al. 2004 [4]	**LVIDd**	1.53	1.27 - 1.85	0.294	1.4*	1.13 - 1.6	< 0.0001*
(*N* = 494)	**LVIDs**	0.95	0.71 - 1.26	0.315	0.93	0.75 - 1.15	0.5682
	**IVSd**	0.41	0.29 - 0.59	0.241	0.46*	0.34 - 0.65	< 0.0001*
	**IVSs**	0.58	0.43 - 0.79	0.240	0.6*	0.49 - 0.93	< 0.0001*
	**LVPWd**	0.42	0.29 - 0.6	0.232	0.48*	0.39 - 0.6	< 0.0001*
	**LVPWs**	0.64	0.48 - 0.87	0.222	0.68*	0.53 - 0.86	0.0153*
Esser et. al. 2020 [10]	**LVIDd**	1.38	1.17 - 1.63	0.322	1.28*	1.05 - 1.53	< 0.0001*
(*N* = 6097)	**LVIDs**	0.87	0.7 - 1.09	0.346	0.87	0.69 - 1.08	1.0000
	**IVSd**	0.36	0.27 - 0.49	0.289	0.41*	0.31 - 0.59	0.0006*
	**IVSs**	0.51	0.38 - 0.68	0.276	0.55*	0.46 - 0.81	0.0002*
	**LVPWd**	0.4	0.3 - 0.53	0.261	0.46*	0.37 - 0.56	< 0.0001*
	**LVPWs**	0.6	0.46 - 0.78	0.247	0.64*	0.48 - 0.81	0.0046*
Visser et al. 2019 [9]	**LVIDd**	1.4	1.2 - 1.64	0.299	1.38	1.12 - 1.63	0.5758
(*N* = 122)	**LVIDs**	0.73	0.55 - 0.96	0.062	0.79*	0.63 - 0.99	0.0006*

*IVSd/s* Interventricular septum diastole/systole, *LVIDd/s* Left ventricular internal diameter diastole/systole, *LVPWd/s* Left ventricular posterior wall diastole/systole, *N* Number of subjects per study, *RI* Reference interval (95% prediction interval)

all parameters in cm; median and RI for pugs are calculated from measurements of the 51 pugs included in the present study, scaled with the corresponding exponents for each publication; comparison of medians with sign test, significant p values highlighted with an asterisk (*).

For left atrial-to-aortic root ratio (LA:Ao), the median of pugs was significantly lower compared to Visser et al. (2019) (*p* = 0.0003) and showed no significant difference to median of Rishniw et al. (2019) (Table 7). Compared to both studies, RI of LA:Ao in pugs was overall similar to RIs of the published studies.

**Table 7:** Comparison of LA:Ao in pugs from the present study to selection of published interbreed references

		**median**	**RI**	
**LA:Ao**				
Pugs	(*N* = 51)	1.3	0.97 - 1.63	
Visser et al. 2019 [9]	(*N* = 122)	1.42*	1.0 - 1.68	*sign. higher than in pugs (p = 0.0046)
Rishniw et al. 2019 [8]	(*N* = 238)	1.34	1.04 - 1.7	no significant difference (p = 0.2624)

*LA:Ao* Left-atrial-to-aortic ratio, *N* Number of subjects per study, *RI* Reference interval (95% prediction interval, 2.5% - 97.5% percentile)

comparison of medians with sign test, significant p values highlighted with an asterisk (*).

On examining the right ventricular (RV) function, tricuspid annular plane systolic motion excursion (TAPSE) values in pugs were significantly lower compared to values of generic interbreed references (*p* < 0.0001; Table 8). The RI of pugs turned out to be lower than RIs of the interbreed study, even when compared to a BW-dependent interbreed RI of 9 kg similar to the mean BW of the present study population (8.9 kg). Prediction intervals of TAPSE and TAPSE indexed to Ao (TAPSE:Ao) appeared to be distinctly lower in pugs. Both TAPSE and TAPSE:Ao showed no significant correlation to BW in pugs in this study.

**Table 8:** Comparison of TAPSE and TAPSE:Ao in pugs in the present study to selected interbreed studies

		**mean/median**	**RI**	
**TAPSE [mm]**				
*Pugs*	(*N *= 40)	9.32	6 - 12.75	
*Visser et al. 2015*	(*N* = 80)	13.37*	11.4 - 15.53	*sign. higher than in pugs (p < 0.0001)
*Visser et al. 2015 (BW-dependent RI for dogs of 9 kg)*	(*N* = 80)	N/A	9.2 - 14.7	not applicable, since data not available
**TAPSE:Ao**				
*Pugs*	(*N* = 40)	0.62	0.38 - 0.81	
*Caivano et al. 2018* *[*42*]*	(*N* = 137)	N/A	0.65 - 1.07	not applicable, since data not available

*BW* Body weight, *RI* Reference interval (95% prediction interval, 2.5% - 97.5% percentile), *N/A* Not available, *TAPSE* Tricuspid annular plane systolic motion excursion in mm, *TAPSE:Ao* TAPSE indexed to aortic root diameter

comparison of medians with sign test, between means with one sample t-test, significant p values highlighted with an asterisk (*).

## Discussion

Echocardiographic reference ranges often derive from a population of various breeds [4, 9, 10, 45, 46]. Even though techniques such as allometric scaling have proven to be useful when establishing interbreed reference ranges [4, 9, 10], they however do not take breed-specific characteristics such as exceptional body conformation or breed-specific diseases into account. Therefore, this study aimed to establish breed-specific echocardiographic reference values for the pug and to reveal influences of independent variables and associations with BOAS severity. Furthermore, a comparison of selected echocardiographic measurements of pugs to values of previous interbreed studies was conducted.

It has to be acknowledged that generating reference ranges from a breed with a generally small population and additionally high prevalence for a systemic disease, like in the pug, leads to a small amount of healthy subjects and limitations regarding the validity of the references. However, including animals irrespective of their clinical status to achieve a higher number of subjects may lead to biased references [43]. As brachycephaly is a breed-characteristic feature, and a high prevalence of BOAS in pugs can be observed [27], BOAS has to be considered in this breed for an echocardiographic study with the aim of establishing reference ranges. We therefore knowingly included pugs presenting with clinical signs of BOAS and performed additional analyses between the different BOAS groups and Grades to determine the possible influence on BOAS on echocardiographic variables.

Moreover, few studies on echocardiography in brachycephalic dogs, and even fewer data for the pug in particular exist [31, 32]. In these former studies, no distinct abnormalities in echocardiography in brachycephalic dogs could be detected. However, significant differences in some echocardiographic measurements between brachycephalic and mesocephalic groups, for example for LV internal diameter in systole (LVIDs) and LA:Ao [31] and Pvel [32] could be determined in the abovementioned studies. These previous findings were further investigated in a larger population of pugs in the current study.

Furthermore, no comparison between different impairments of BOAS was conducted in the two above-mentioned studies, and pugs were either not examined [31] or underrepresented (three pugs out of 20 examined brachycephalic dogs) [32].

However, chronic obstructive respiratory diseases such as BOAS may also lead to PH [28, 29]. PH is a cardiovascular disease measured by pulmonary artery pressure (PAP), ultimately leading to valve insufficiencies and changes in the right heart, for example in TAPSE [28, 30, 45]. Thus, the present study focusing on pugs could help cardiologists in distinguishing pathological changes due to primary cardiac disease from changes related to BOAS.

In the present study, prediction intervals for the entire study population were uniformly generated by using the sample quantiles for each parameter, no matter if normally distributed or not, and 90% confidence intervals using bootstrap method were calculated [43]. These may serve as general reference intervals for pugs. In non-normally distributed variables, the median and range can be taken into account for optimally evaluating measurements in individual cases. Multiple linear regression equations for all echocardiographic parameters, in which at least one independent variable had significant impact, and the corresponding adjusted R-squares (R^2^) were calculated. As R^2^ is one of several possible criteria for interpreting ‘goodness of fit’ of the model [47], it may help with interpreting the impact of the independent variables on each echocardiographic parameter.

Also, the study population herein was subdivided into groups and Grades of BOAS [26, 27] for comparing demographic data and echocardiographic measurements with regard to different clinical severities of BOAS. When examining demographic values, no significant differences could be detected between the BOAS groups or Grades regarding age, BS, BW, HR and SAP. Female and male pugs were equally distributed in the BOAS groups and both at least represented in all BOAS Grades. Under these conditions, regarding the echocardiographic measurements of the BOAS- and BOAS+ groups, no significant differences could be detected, except for Pvel being significantly lower in the BOAS+ group. When subdividing pugs even further into Grades 0 to 3, no significant differences between the Grades were detected. Still, a decrease in Pvel could be observed, the higher the BOAS Grade. Lack of statistical significant difference most likely resulted from a small amount of subjects per BOAS Grade and was therefore only detected in Pvel when combining the Grades in the subgroups BOAS- and BOAS+.

The findings in the Doppler examination of the right ventricular outflow tract indicated that pulmonic velocity declined with increasing severity of BOAS. Due to a lack of statistical difference regarding BOAS Grades, it is possible that this finding is unrelated to BOAS and might be mere chance. However, lower pulmonic velocities in brachycephalic dogs than in control dogs have already been described in a previous study [32]. Therein, 20 brachycephalic dogs were examined, however without comparable clinical BOAS Grading, including 12 Shi Tzus, four French Bulldogs, three pugs and one English Bulldog. This aforementioned study found no evidence of changes in PAP in dogs with Brachycephalic Syndrome and no explanation for lower pulmonic velocities. In another study, 16 brachycephalic dogs (four pugs and 12 French Bulldogs) underwent rhinoplasty, and RV function as well as pulmonic velocity and pressure gradient between pulmonary artery and right ventricle was examined via echocardiography before and after surgery [48]. This study revealed a significant increase in Pvel and a change in pressure gradient after rhinoplasty, and they hypothesised this may be due to a reduction of the upper airway obstruction and consequently a decrease of pulmonary vascular resistance. Values of both studies, as well as our measurements in pugs, are still within reported reference intervals for dogs for Pvel (0.6 - 1.8 m/s, [49]), except for the lower range limit in BOAS Grade 3 (0.54 m/s) being slightly lower than the stated reference interval. However, regarding the findings of both the aforementioned studies as well as of the present study, lower Pvel values in more severely BOAS-affected dogs may be a first echocardiographic indication of increased pulmonary vascular resistance and a rising PAP. Thus, clinically affected pugs may be at a higher risk to develop pathological changes in the cardiorespiratory system, such as PH.

Since BOAS has been described as possibly leading to PH [28, 29], measurable changes in the form of a rising PAP would be assumed in cases where PH develops. Since only patients with cardiac health identified on 2-D Mode, M-Mode, and Colour Doppler were included in this study, patients with signs of PH would have been excluded beforehand and showed at most very early stages of PH. Thus, common techniques for estimating PAP to check for differences between the BOAS Grades could not be resorted to [30, 50], and no direct connections between severity of BOAS and PH could be identified in the present study.

However, apart from this, right ventricular systolic dysfunction is another indicator for PH [30]. For evaluating RV systolic function in standard echocardiography, TAPSE has proven itself to be reproducible and easily obtainable [41, 45]. Differences in TAPSE between healthy and non-healthy subjects of different cardiac diseases or different stages of diseases in dogs have been widely detected [45, 51–53]. TAPSE has also been investigated as a predictor for PH; it decreases significantly depending on severity of PH and it shows poor sensitivity but good specificity [45]. Similar findings could be observed for the indexed TAPSE:Ao [42]. Thus, an early reduced RV function could be more pronounced in pugs with higher BOAS Grades, detectable through alterations in TAPSE. However, no significant differences between BOAS- and BOAS+ or BOAS Grades in pugs could be found in the present study. This is in accordance with findings of the study by Hainfellner et al. (2021), in which there was no significant change in TAPSE before compared to after rhinoplasty [48]. When further comparing measurements of TAPSE in pugs to those of interbreed references, overall, lower TAPSE and TAPSE:Ao values were detected in pugs than in previous studies of various breeds, even when compared to an RI of a similar BW [41, 42]. Generally low TAPSE and TAPSE:Ao in pugs may therefore be breed-characteristic, as we could not detect any relation between a reducing RV function and an increasing clinical BOAS Grade.

In conclusion, all echocardiographic parameters, except for Pvel, showed no significant differences between the BOAS groups or Grades. This may be a first indication that BOAS could become clinically relevant in more severe cases. However, clinical impairment by BOAS does not seem to have a major impact on echocardiographic measurements and reference intervals for pugs can be used regardless of their BOAS status.

Echocardiographic measurements in pugs significantly deviated from interbreed values in most cases. Regarding the M-Mode measurements, they had significantly lower medians of LV internal dimensions at end-diastole and end-systole compared to two previous interbreed studies [4, 10]. Regarding LVIDd, throughout all compared studies, a noticeable number of pugs were even found to lie below the studies lower reference limits thereof [4, 9, 10]. The median IVSd and IVSs of pugs in the present study were also found to be significantly thicker compared to Cornell et al. (2004) and Esser et al. (2020). Noteworthy is that these significant deviations in reference intervals appeared even though measurements of pugs have already been normalised with the equivalent allometric equations of each respective interbreed study. Even though differences were statistically significant, they were mostly still minor, and possibly clinically irrelevant. Nevertheless, the findings of the present study underline the utility of breed-specific references.

Moreover, LA:Ao in the present study was found to be overall similar to values of recent interbreed studies. Nonetheless, both more recent interbreed studies [8, 9] and breed-specific studies [15, 16, 54] frequently reported higher LA:Ao reference values. These often exceeded the cut off value of ≥ 1.6 specified in the current consensus statement for classifying dogs with myxomatous mitral valve disease [2], which was based on a study in Cavalier King Charles Spaniels [6]. Similar findings could be detected for pugs in the present study, with the upper RI of LA:Ao exceeding 1.6. This suggests that the recommended value for LA:Ao may not be equally applicable, as it is based on a specific breed, and breed-specific values for LA:Ao for pugs should be considered in echocardiographic examination.

The present study has some limitations: for establishing reference ranges, a sample size of ≥ 120 subjects has been described as ideal by the American Society of Veterinary Clinical Pathology [43]. Still, a sample size of > 40 is seen as adequate for veterinary studies on specific breeds, as a small number of animals of the specific breed may be available for examination. Due to this, comparable sample sizes are common in breed-specific studies in small animal cardiology [7, 13, 16, 55]. Nonetheless, and especially due to the subdivision into BOAS Grades and groups in this study population of pugs, reference values should be interpreted with caution.

Even though intra- and interobserver agreement have already been investigated for most echocardiographic values, some differences between observers may always occur in echocardiography [56, 57]. To ensure optimal evaluation and comparability, all measurements were carried out in accordance with recent guidelines and with a standardised echocardiographic protocol for each subject.

## Conclusion

Echocardiographic reference ranges for pugs were generated. Pugs were subdivided into groups and Grades depending on the presence of signs of BOAS. Significant lower Pvel was observed for the group of pugs clinically affected by BOAS. Apart from this, BOAS did not show any significant influence on echocardiographic measurements. Differences in comparison to reference values of various breeds were detected for various parameters such as LV internal dimensions and LV wall thickness, and TAPSE.

## Supplementary Information

**Additional file 1.** 

**Additional file 2.**

## Data Availability

The dataset used during the current study is available from the corresponding author on reasonable request.

## Abbreviations

2-D: two-dimensional
LVESV: left ventricular end-systolic volume
Ao: Aortic root diameter
LVID: left ventricular internal diameter
Avel: aortic peak velocity
LVL: left ventricular length
BOAS: Brachycephalic Obstructive Airway Syndrome
LVPW: Left ventricular posterior wall
BW: body weight
LVW: left ventricular width
BS: Body surface area
MV A/E: mitral A/E-wave
CI: Confidence interval
PAP: Pulmonary artery pressure
d: Diastole
PH: Pulmonary hypertension
EDV:BW: Left ventricular end-diastolic volume indexed to BW
Pvel: Pulmonic peak velocity
EDVI: Indexed left ventricular end-diastolic volume to BS
R^2^: Adjusted r-square
EF: Ejection fraction
RI: Reference interval
ESV:BW: Left ventricular end-systolic volume indexed to BW
ESVI: indexed left ventricular end-systolic volume to BS
RV: Right ventricular
ET: Exercise test
RV: Right ventricular
EPSS: E-point-to-septal-separation
s: Systole
FS: Fractional shortening
SAP: Systolic arterial pressure
HR: Heart rate
SD: Standard deviation
IVS: Interventricular septum
SI: Sphericity index
LA: Left atrial diameter
SMOD: Simpson’s modified method of discs
LA:Ao: Left-atrial-to-aortic-root diameter
TAPSE: Tricuspid annular plane systolic motion excursion
LV: Left ventricle
TAPSE:Ao: TAPSE indexed to aortic root diameter
LVEDV: left ventricular end-diastolic volume
TV A/E: tricuspid A/E-wave
