# Supplementary material for: Reference ranges for standard-echocardiography in pugs and impact of clinical severity of Brachycephalic Obstructive Airway Syndrome (BOAS) on echocardiographic parameters
Source: BMC Vet Res. 2022 Jul 20;18:282. doi: 10.1186/s12917-022-03348-8 (PMC9297600; doi:10.1186/s12917-022-03348-8)
Supplement: Supplementary file 1 — Additional file 1. [file 12917_2022_3348_MOESM1_ESM.docx]

**Additional file 1 - Material and Methods**

**Functional grading system of BOAS based on respiratory signs before and after an exercise test (ET)**

|  |  | **Respiratory noise^a^** | **Inspiratory effort^b^** | **Dyspnea/Cyanosis/Syncope^c^** |
| --- | --- | --- | --- | --- |
| **Grade 0** | Pre-ET | Not audible | Not present | Not present |
|  | Post-ET | Not audible | Not present | Not present |
| **Grade 1** | Pre-ET | Not audible or mild | Not present | Not present |
|  | Post-ET | Mild | Not present to mild | Not present |
| **Grade 2** | Pre-ET | Mild to moderate | Mild to moderate | Not present |
|  | Post-ET | Moderate to severe | Moderate to severe | Mild dyspnea; cyanosis or syncope not present |
| **Grade 3** | Pre-ET | Moderate to severe | Moderate to severe | Moderate to severe dyspnea; may or may not present cyanosis; inability to exercise |
|  | Post-ET | Severe | Severe | Severe dyspnea; may or may not present cyanosis or syncope |

The clinical grading was based on respiratory signs before (pre-ET) and after an exercise test (post-ET) corresponding to Liu et al 2015 (1) and modified to match the present study design: Dogs underwent a submaximal ET in an individual trotting pace on a treadmill and post-ET grading was conducted immediately after 5 minutes of exercise.

^a^Respiratory noise was diagnosed by pharyngolaryngeal and thoracal auscultation. Mild: only audible under auscultation; moderate: intermittent audible noise that can be heard without stethoscope; severe: constant audible noise that can be heard without stethoscope.

^b^An abnormal respiratory cycle characterized by evidence of increased effort to inhale the air with the use of diaphragm and/or accessary muscles of respiration and/or nasal flaring with an increase in breathing rate. Mild: regular breathing patterns with minimal use of diaphragm; moderate: evidence of use of diaphragm and accessary muscles of respiration; severe: marked movement of diaphragm and accessary muscles of respiration.

^c^Dogs that have had episodes of syncope and/or cyanosis as documented by owner's report are classified into Grade 3 without ET. Mild dyspnea: presents sign of discomfort; moderate dyspnea: irregular breathing, signs of discomfort; severe dyspnea: irregular breathing with sings of breathing discomfort and difficulty in breathing.

**Echocardiographic measurements**

#### 2D measurements

From a 2-D right parasternal short-axis view, at the level of the aortic valve and with the left atrium showing a tear drop shape, left atrial diameter (LA) and aortic root diameter (Ao) were measured in early ventricular diastole, on the first frame of aortic valve closure. For measuring Ao, a line from the midpoint of the convex curvature of the wall of the right aortic sinus to the point where aortic wall and non-coronary and left coronary aortic cusp merge was drawn. The line extending the Ao line to the blood tissue interface of the LA wall formed the LA diameter. If a pulmonary vein prohibited elongation by entering the LA at the particular measurement point, the caliper was placed either on an extrapolation of the atrial border or the measurement point was chosen medial or lateral to the vein, putting a slight offset to the desired elongation (2). LA:Ao was calculated.

From a 2-D right parasternal long-axis four chamber view, end-diastolic length (LVLd) and width (LVWd) of the left ventricle (LV) were obtained. LVLd was measured from the middle of a line connecting the annuli of the mitral valve to the endocardial border of the LV apex. LVWd was obtained measuring the widest distance between endocardial borders of the LV free wall and the interventricular septum. LVLd to LVWd, the sphericity index (SI), was then calculated.

For assessment of left ventricular function, monoplane Simpson’s modified method of discs (SMOD)-derived left ventricular end-diastolic volume (LVEDV) and end-systolic volume (LVESV) were calculated from images obtained from the right parasternal long-axis four-chamber view. If the apex of the heart could not be visualized completely, images from the left apical 4-chamber view were used. End-diastolic (when LV was at its largest, pursuant to or directly after the closure of the mitral valve) and end-systolic state (the last frame before mitral valve opening) were determined via frame-by-frame analysis (3). Maximum LV length was defined as the distance measured from the middle of a line connecting the two opposite annuli of the mitral valve to the endocardial border of the LV apex, forming the most distant point of the LV contour. By tracing the endocardial border, LV area was measured and LVEDV, LVESV were automatically calculated. Ejection fraction (EF) (%) was calculated as: (LVEDV−LVESV)/LVEDV x 100. By indexing both measurements to BS, EDVI and ESVI were computed. By indexing both measurements to BW, EDV:BW and ESV:BW were computed (4).

#### M-Mode measurements

In accordance with the recommendations of the American Society for Echocardiography (5), following parameters from M-mode images of the right parasternal short-axis view using the leading edge to leading edge method were obtained: interventricular wall thickness (IVS), LV posterior wall thickness (LVPW), and LV internal diameter (LVID), each in systole (s) and diastole (d). Fractional shortening (FS) (%) = (LVIDd-LVIDs)/LVIDd x 100 was calculated. From the right parasternal long-axis M-mode, E-point-to-septal-separation (EPSS) was measured at the level of the mitral valve.

Tricuspid annular plane systolice motion excursion (TAPSE) was obtained in M-Mode from left apical 4-chamber view, with the image adjusted for the lateral portion of the tricuspid annulus (6 - 8). The M-Mode cursor was aligned as parallel as possible to the right ventricular free wall, capturing the longitudinal displacement of the tricuspid valve plane. TAPSE was then measured as the distance between the most basilar positions of the annulus at end-diastole to its most apical displacement at end-systole, using the leading edge method. Highest measurable value was recorded to avoid underestimation. TAPSE:Ao was calculated (8).

#### Doppler assessment

Via continuous and pulse wave Doppler, maximum aortic velocity (Avel), pulmonic velocity (Pvel), and if measurable, jet velocities of valve regurgitations were measured. Avel was obtained from a subcostal view (9), the alignment of the cursor perpendicular to the aortic blood flow, verified by foregoing colour Doppler flow. If correct alignment could not be generated, Avel was measured from the left apical five-chamber view in the exact same manner. Pvel was measured from the right parasternal short-axis and left apical cranial view, whereof the highest velocity measureable was recorded to avoid underestimation.

Atrioventricular valves were screened for morphological changes and presence of prolapse using both 2-D and colour Doppler in right parasternal long-axis and left apical four-chamber view. If a mitral valve regurgitation (MR) was present, the ratio of the regurgitant jet area to the area of the affected atrium (%) was subjectively assessed. MR was considered trivial or clinically non-significant if the regurgitant jet area to left atrium area was < 15%. Regurgitations with a ratio of 15 - 30% were considered mild, 30 - 50% moderate and > 50% severe (10, 11). Tricuspid valve regurgitations (TR) with low jet velocities and only trivial or mild in colour Doppler were considered as non-pathological and therefore clinically non-significant. Aortic and pulmonic valves were screened for abnormalities in the same manner. Trivial to mild aortic (AR) or pulmonic regurgitations (PR) were considered clinically non-significant.

Early (E) and late (A) ventricular filling velocities were assessed in left-apical 4-chamber view via pw-Doppler, by placing the cursor between and slightly caudal to the mitral or tricuspid leaflets. Mitral A- and E- wave (MV A, MV E) and Tricuspid A- and E- wave (TV A, TV E) were then used to determine E-to-A velocity ratio (MV E:A, TV E:A).

1. Liu NC, Sargan DR, Adams VJ, Ladlow JF. Characterisation of Brachycephalic Obstructive Airway Syndrome in French Bulldogs Using Whole-Body Barometric Plethysmography. PloS one. 2015;10(6):e0130741.

2. Hansson K, Haggstrom J, Kvart C, Lord P. Left atrial to aortic root indices using two-dimensional and M-mode echocardiography in cavalier King Charles spaniels with and without left atrial enlargement. Vet Radiol Ultrasound. 2002;43(6):568-75.

3. Lang RM, Bierig M, Devereux RB, Flachskampf FA, Foster E, Pellikka PA, et al. Recommendations for chamber quantification: a report from the American Society of Echocardiography's Guidelines and Standards Committee and the Chamber Quantification Writing Group, developed in conjunction with the European Association of Echocardiography, a branch of the European Society of Cardiology. J Am Soc Echocardiogr. 2005;18(12):1440-63.

4. Wess G, Bauer A, Kopp A. Echocardiographic reference intervals for volumetric measurements of the left ventricle using the Simpson's method of discs in 1331 dogs. Journal of veterinary internal medicine. 2021;35(2):724-38. doi: https://doi.org/10.1111/jvim.16089.

5. Lang RM, Badano LP, Mor-Avi V, Afilalo J, Armstrong A, Ernande L, et al. Recommendations for cardiac chamber quantification by echocardiography in adults: an update from the American Society of Echocardiography and the European Association of Cardiovascular Imaging. J Am Soc Echocardiogr. 2015;28(1):1-39.e14.

6. Pariaut R, Saelinger C, Strickland KN, Beaufrere H, Reynolds CA, Vila J. Tricuspid annular plane systolic excursion (TAPSE) in dogs: reference values and impact of pulmonary hypertension. J Vet Intern Med. 2012;26(5):1148-54.

7. Poser H, Berlanda M, Monacolli M, Contiero B, Coltro A, Guglielmini C. Tricuspid annular plane systolic excursion in dogs with myxomatous mitral valve disease with and without pulmonary hypertension. J Vet Cardiol. 2017;19(3):228-39.

8. Caivano D, Dickson D, Pariaut R, Stillman M, Rishniw M. Tricuspid annular plane systolic excursion-to-aortic ratio provides a bodyweight-independent measure of right ventricular systolic function in dogs. J Vet Cardiol. 2018;20(2):79-91.

9. Deinert M, Janthur M, G. Kresken J, Schneider M, Tobias R, Wendt R. Standardisierter kardiologischer Untersuchungsgang beim Hund mit zentraler Datenerfassung des Collegium Cardiologicum (CC) e. V. Tierarztl Prax Ausg K Kleintiere Heimtiere. 2012;40:283-9.

10. Giraut S, Haggstrom J, Koskinen LLE, Lohi H, Wiberg M. Breed-specific reference ranges for standard echocardiographic measurements in salukis. J Small Anim Pract. 2019;60(6):374-8.

11. Zoghbi WA, Enriquez-Sarano M, Foster E, Grayburn PA, Kraft CD, Levine RA, et al. Recommendations for evaluation of the severity of native valvular regurgitation with two-dimensional and Doppler echocardiography. J Am Soc Echocardiogr. 2003;16(7):777-802.
