# Supplementary material for: Reference ranges for standard-echocardiography in pugs and impact of clinical severity of Brachycephalic Obstructive Airway Syndrome (BOAS) on echocardiographic parameters
Source: BMC Vet Res. 2022 Jul 20;18:282. doi: 10.1186/s12917-022-03348-8 (PMC9297600; doi:10.1186/s12917-022-03348-8)
Supplement: Supplementary file 2 — Additional file 2. [file 12917_2022_3348_MOESM2_ESM.docx]

**Additional file 2 – Results**

*Table a: Mean ± SD of demographic data for 42 pugs allocated to BOAS Grades*

|  | **Grade 0** | **Grade 1** | **Grade 2** | **Grade 3** | **p value** |
| --- | --- | --- | --- | --- | --- |
| N = 42 | 13 | 8 | 15 | 6 |  |
| **age [years]** | 4.2 ± 1.4 | 3.9 ± 2.8 | 4.6 ± 2.3 | 4.6 ± 2.1 | 0.6864 |
| **BW [kg]** | 9.1 ± 1.2 | 8.9 ± 1.2 | 9.0 ± 1.3 | 7.8 ± 1.5 | 0.2927 |
| **BS [m²]** | 0.44 ± 0.04 | 0.43 ± 0.04 | 0.44 ± 0.04 | 0.39 ± 0.05 | 0.2431 |
| **HR [bpm]** | 103 ± 22 | 113 ± 20 | 115 ± 22 | 108 ± 22 | 0.5620 |
| **SAP [mmHg]** | 114.5 ± 6.8 | 124.4 ± 16.3 | 122.1 ± 10.4 | 118.0 ± 17.3 | 0.1876 |
| **sex** |  |  |  |  |  |
| **male (n/i)** | 7 (1/6) | 3 (0/3) | 8 (1/7) | 2 (1/1) | 0.7557 |
| **female (n/i)** | 6 (1/5) | 5 (1/4) | 7 (3/4) | 4 (2/2) |  |

BOAS, Brachycephalic Obstructive Airway Syndrome; bpm, beats per minute; BS, body surface area; BW, body weight; HR, heart rate, i, intact; N, number of subjects; n, neutered; SAP, systolic arterial pressure

Note: BOAS functional grading system according to Liu et al. 2015, modified to present study design. P values from comparsion of BOAS- to BOAS+ group by using Wilcoxon rank-sum test.

*Table b: Multiple linear regression analysis models of echocardiographic variables in 51 pugs*

| **dependent variable** | **multiple linear regression equation** | **R²** |
| --- | --- | --- |
| LVLd | 2.059 + 0.248 x BW | 0.56 |
| LVWd | 1.616 + 0.112 x BW | 0.3 |
| LVEDV | 1.328 + 1.950 x BW | 0.37 |
| LVESV | 11.039 - 0.033 x HR | 0.08 |
| ESV:BW | 1.232925 - 0.0036HR | 0.07 |
| ESVI | 25.247 - 0.075 x HR | 0.08 |
| EF | 24.105 + 1.836 x BW + 0.183 x HR | 0.21 |
| LVIDd | 1.629 + 0.114 x BW | 0.24 |
| LVIDs | 1.242 + 0.069 x BW | 0.1 |
| IVSd | 0.407 + 0.042 x BW | 0.13 |
| IVSs | 0.732 + 0.035 x BW | 0.07 |
| LVPWs | 0.803 + 0.034 x BW | 0.1 |
| LA | 0.390 + 0.181 x BW | 0.42 |
| Ao | 0.973 + 0.060 x BW | 0.23 |
| LA:Ao | 0.719 + 0.068 x BW | 0.26 |
| Pvel | 0.428 + 0.003 x HR | 0.21 |
| MV E | 0.883 - 0.001 x age | 0.07 |
| MV A | 0.359 + 0.002 x HR + 0.056 x sex | 0.25 |
| MV E:A | 1.932 - 0.004 x HR - 0.003 x age | 0.11 |
| TV E | 0.497 + 0.031 x BW - 0.002 x age | 0.14 |
| TV A | 0.327 + 0.002 x HR - 0.001 x age + 0.084 x sex | 0.3 |
| TV E:A | 0.397 + 0.116 x BW | 0.2 |
|  |  |  |

R², adjusted r-squared values; age, age in months; Ao, aortic root diameter; BW, body weight in kg; EF, ejection fraction; ESVI, indexed left ventricular end-systolic volume; HR, heart rate; IVSd/s, interventricular septum diastole/systole; LA, left atrial diameter; LVEDV, left ventricular end-diastolic volume; LVESV; left ventricular end-systolic volume; LVIDd/s, left ventricular internal diameter diastole/systole, LVLd, left ventricular length diastole; LVPWs, left ventricular posterior wall systole; LVWd, left ventricular width diastole; MV A, mitral A-wave; MV E, mitral E-wave; Pvel, pulmonic peak velocity; sex, male = 0, female = 1; TV A, tricuspid A-wave; TV E, tricuspid E-wave

*Table c: 2-D measurements and SMOD-derived LV volumes subdivided into BOAS Grade 0 to 3*

| **Grade** | **N** | **variable** | **mean** | **median** | **SD** | **min** | **max** |
| --- | --- | --- | --- | --- | --- | --- | --- |
| 0 | 13 | LA Ao LA:Ao LVLd LVWd SI EDV ESV EDVI ESVI EDV:BW ESV:BW EF | 2.13 1.54 1.39 4.39 2.66 1.68 19.97 7.44 45.51 17.08 2.21 0.83 62.77 | 2.21 1.53 1.44 4.36 2.72 1.61 19.95 7.53 44.4 16.67 2.22 0.75 61.32 | 0.37 0.12 0.19 0.39 0.25 0.18 4.0 2.26 8.94 5.81 0.46 0.31 8.72 | 1.33 1.31 0.95 3.83 2.14 1.33 13.07 3.7 28.11 8.73 1.32 0.43 45.39 | 2.69 1.7 1.63 5.26 2.93 1.93 26.33 11.26 57.71 29.1 2.76 1.5 74.51 |
| 1 | 8 | LA Ao LA:Ao LVLd LVWd SI EDV ESV EDVI ESVI EDV:BW ESV:BW EF | 1.96 1.48 1.31 4.11 2.61 1.59 17.82 7.37 41.39 17.31 2.03 0.85 58.19 | 1.88 1.5 1.3 4.04 2.52 1.61 16.69 6.01 40.38 13.62 1.93 0.67 60.48 | 0.36 0.14 0.16 0.39 0.29 0.16 4.75 2.75 11.09 7.08 0.57 0.37 12.46 | 1.38 1.22 1.1 3.61 2.27 1.3 11.63 4.74 26.61 10.97 1.29 0.52 35.9 | 2.56 1.66 1.62 4.89 2.97 1.8 23.64 11.69 57.56 28.46 2.88 1.43 73.7 |
| 2 | 15 | LA Ao LA:Ao LVLd LVWd SI EDV ESV EDVI ESVI EDV:BW ESV;BW EF | 1.95 1.52 1.28 4.37 2.62 1.68 18.93 7.44 43.19 17.09 2.1 0.83 60.37 | 1.92 1.5 1.23 4.2 2.62 1.65 18.45 7.3 41.97 16.79 2.05 0.84 59.16 | 0.33 0.15 0.18 0.49 0.28 0.2 3.63 1.98 6.34 4.28 0.31 0.22 8.24 | 1.51 1.35 0.97 3.65 2.23 1.34 14.03 3.41 33.62 7.09 1.67 0.33 50.24 | 2.56 1.95 1.61 5.22 3.28 2.17 27.25 11.99 52.46 25.7 2.63 1.31 82.66 |
| 3 | 6 | LA Ao LA:Ao LVLd LVWd SI EDV ESV EDVI ESVI EDV:BW ESV:BW EF | 2.01 1.54 1.31 3.91 2.46 1.61 15.32 6.51 38.61 16.62 1.98 0.86 56.8 | 2.17 1.54 1.27 3.73 2.61 1.58 15.6 6.95 40.68 16.66 1.93 0.81 57.89 | 0.4 0.26 0.14 0.47 0.35 0.23 4.19 1.47 8.07 4.04 0.42 0.24 6.03 | 1.42 1.25 1.14 3.49 1.95 1.33 9.5 4 27.57 11.61 1.51 0.63 49.81 | 2.39 1.92 1.53 4.63 2.83 1.91 19.87 7.6 46.78 22.79 2.6 1.27 62.55 |

2-D, two dimensional; Ao, aortic root diameter; EF, ejection fraction; EDV:BW, left ventricular end-diastolic volume indexed to body weight; EDVI, indexed left ventricular end-diastolic volume to body surface area; ESV:BW, left ventricular end-systolic volume indexed to body weight; ESVI, indexed left ventricular end-systolic volume to body surface area; LA, left atrial diameter; LV, left ventricle; LVEDV, left ventricular end-diastolic volume; LVESV; left ventricular end-systolic volume; LVLd, left ventricular length diastole; LVWd, left ventricular width diastole; N, number of subjects; SI, sphericity index; SD, standard deviation; SMOD, Simpson’s modified method of discs

*Table d: M-Mode measurements subdivided into BOAS Grade 0 to 3*

| **Grade** | **N** | **variable** | **mean** | **median** | **SD** | **min** | **max** |
| --- | --- | --- | --- | --- | --- | --- | --- |
| 0 | 13  12 | LVIDd LVIDs IVSd IVSs LVPWd LVPWs FS EPSS  TAPSE TAPSE:Ao | 2.67 1.82 0.81 1.04 0.80 1.13 30.69 2.62  9.49 0.62 | 2.71 1.84 0.79 1.04 0.82 1.11 30.69 2.60  9.45 0.62 | 0.15 0.31 0.13 0.13 0.08 0.12 6.38 0.99  1.48 0.09 | 2.41 1.02 0.57 0.82 0.66 0.89 17.43 1.40  7.10 0.47 | 2.89 2.23 1.09 1.25 0.97 1.41 41.22 5.00  12.10 0.75 |
| 1 | 8  5 | LVIDd LVIDs IVSd IVSs LVPWd LVPWs FS EPSS  TAPSE TAPSE:Ao | 2.68 1.88 0.74 1.014 0.80 1.14 29.57 3.99  9.70 0.62 | 2.67 1.90 0.72 1.02 0.77 1.13 29.84 2.85  9.90 0.61 | 0.31 0.28 0.11 0.13 0.13 0.16 7.60 2.84  0.89 0.05 | 2.11 1.46 0.62 0.87 0.66 0.89 18.71 1.30  8.20 0.55 | 3.10 2.24 0.94 1.19 1.02 1.40 40.40 10.10  10.50 0.67 |
| 2 | 15  12 | LVIDd LVIDs IVSd IVSs LVPWd LVPWs FS EPSS  TAPSE TAPSE:Ao | 2.61 1.84 0.83 1.10 0.82 1.08 29.56 3.02  9.69 0.64 | 2.61 1.82 0.82 1.11 0.84 1.06 29.05 3.00  8.90 0.6 | 0.24 0.27 0.21 0.20 0.09 0.13 6.60 1.28  1.88 0.12 | 2.19 1.46 0.60 0.82 0.66 0.86 22.82 0.90  6.80 0.5 | 3.19 2.43 1.28 1.48 0.94 1.34 44.86 6.10  12.10 0.08 |
| 3 | 6  4 | LVIDd LVIDs IVSd IVSs LVPWd LVPWs FS EPSS  TAPSE TAPSE:Ao | 2.42 1.83 0.80 0.97 0.78 0.93 24.36 2.97  10.43 0.69 | 2.42 1.83 0.77 0.97 0.74 1.01 23.68 2.85  10.20 0.75 | 0.32 0.25 0.19 0.12 0.08 0.25 3.79 1.65  2.38 0.15 | 1.94 1.50 0.55 0.82 0.72 0.44 19.09 0.70  7.90 0.48 | 2.78 2.21 1.04 1.14 0.91 1.12 30.36 4.90  13.40 0.81 |

Ao, aortic root diameter; EPSS, E-Point to septum separation; FS, fractional shortening; IVSd/s, interventricular septum diastole/systole; LVIDd/s, left ventricular internal diameter diastole/systole; LVPWd/s, left ventricular posterior wall diastole/systole; N, number of subjects; SD, standard deviation; TAPSE, tricuspid annular plane systolic motion excursion

*Table e: Doppler measurements subdivided into BOAS Grade 0 to 3*

| **Grade** | **N** | **variable** | **mean** | **median** | **SD** | **min** | **max** |
| --- | --- | --- | --- | --- | --- | --- | --- |
| 0 | 13 | Avel Pvel MV E MV A MV E:A TV E TV A TV E:A | 1.37 0.85 0.81 0.63 1.33 0.680 0.44 1.69 | 1.31 0.84 0.82 0.59 1.36 0.70 0.47 1.46 | 0.26 0.21 0.11 0.17 0.25 0.14 0.11 0.83 | 0.92 0.51 0.62 0.48 0.61 0.43 0.18 0.90 | 1.75 1.17 1.00 1.15 1.67 0.86 0.60 3.94 |
| 1 | 8 | Avel Pvel MV E MV A MV E:A TV E TV A TV E:A | 1.45 0.87 0.84 0.64 1.32 0.75 0.59 1.34 | 1.450 0.87 0.83 0.69 1.31 0.74 0.58 1.30 | 0.27 0.08 0.14 0.08 0.25 0.08 0.13 0.41 | 1.04 0.75 0.65 0.48 0.93 0.63 0.43 0.84 | 1.84 1.02 1.08 0.71 1.71 0.90 0.75 2.09 |
| 2 | 15 | Avel Pvel MV E MV A MV E:A TV E TV A TV E:A | 1.46 0.78 0.84 0.59 1.43 0.72 0.47 1.55 | 1.47 0.78 0.85 0.58 1.47 0.69 0.47 1.49 | 0.21 0.09 0.12 0.08 0.28 0.15 0.09 0.34 | 1.09 0.59 0.64 0.43 1.03 0.48 0.35 0.79 | 1.75 0.92 1.07 0.73 2.02 1.14 0.71 2.38 |
| 3 | 6 | Avel Pvel MV E MV A MV E:A TV E TV A TV E:A | 1.17 0.72 0.77 0.59 1.33 0.67 0.56 1.24 | 1.12 0.70 0.72 0.58 1.15 0.62 0.61 1.11 | 0.21 0.15 0.18 0.07 0.42 0.19 0.11 0.36 | 0.97 0.54 0.64 0.52 1.07 0.49 0.43 0.79 | 1.51 0.90 1.13 0.70 2.17 0.96 0.68 1.69 |

Avel, aortic peak velocity; MV A, mitral A-wave, MV E, mitral E-wave; N, number of subjects; Pvel, pulmonic peak velocity; SD, standard deviation; TV A, tricuspid A-wave; TV E, tricuspid E-wave

*Table f: Percentage of pugs outside (above or below) the RI of each interbreed publication*

|  | **LVIDd** | |  | **LVIDs** | |  | **IVSd** | |  | **IVSs** | |  | **LVPWd** | |  | **LVPWs** | |
| --- | --- | --- | --- | --- | --- | --- | --- | --- | --- | --- | --- | --- | --- | --- | --- | --- | --- |
| N = 51 | ↑% | ↓% |  | ↑% | ↓% |  | ↑% | ↓% |  | ↑% | ↓% |  | ↑% | ↓% |  | ↑% | ↓% |
| Cornell et al. | - | 15.7 |  | - | 2 |  | 9.8 | - |  | 35.3 | - |  | 2 | - |  | - | 2 |
| Esser et al. | - | 19.6 |  | 2 | 2 |  | 19.6 | - |  | 5.9 | - |  | 11.8 | - |  | 7.8 | 2 |
| Visser et al. | 2 | 9.8 |  | - | 2 |  | N/A | N/A |  | N/A | N/A |  | N/A | N/A |  | N/A | N/A |
|  |  |  |  |  |  |  |  |  |  |  |  |  |  |  |  |  |  |

IVSd/s, interventricular septum diastole/systole; N/A, not available; LVIDd/s, left ventricular internal diameter diastole/systole; LVPWd/s, left ventricular posterior wall diastole/systole; RI, reference interval

Note: N = number of pugs; ↑% = percentage of measurements, normalized corresponding to each publication, above (or below = ↓%) the interbreed RI of each publication; deviations of more than 10% are highlighted in orange, more than 25% in red.
